# Middlesex Hospital

**Published:** 1831-07

**Authors:** 


### HOSPITAL REPORTS.

### MIDDLESEX HOSPITAL.

### *Femoral Aneurysm; Ligature of the Artery.*

Mary Lewis, ætat. fifty-four, was admitted into the Middlesex Hospital, under the care of Mr. Mayo, on the 19th of May. Two months before, she had missed a step, at the bottom of a flight of stairs, down which she was carrying a pail of water: she fell with violence, and the right leg was bent under her. The chief pain, which she remembered having suffered at the time of the accident, was above the knee, about the place where the femoral artery enters the sheath formed by the triceps: she felt something give way at this part; the pain spread from thence over the inner surface of the knee and leg. About three weeks after the accident, she observed that a small tumor had formed at the part which had been so strained.

At the period of the admission of this patient, there was a strongly pulsating aneurysm, as large as a middle-sized orange, at the lower and inner part of the thigh. She was in good health; but she suffered severe and continual pain, accompanied with tenderness on pressure, in the course of the saphenus nerve.

Mr. Mayo, who had seen her previously to her admission, tied the femoral artery the day following, at the point where it passes behind the sartorius. The instant the artery was tied, the severity of the pain along the leg and instep was relieved. The progress of the case has been favorable: the ligature came away on the twenty-second day after the operation. The wound is now all but entirely closed: the tumor has diminished to a third of its original size; and of the pain nothing remains but a slight degree of numbness in the course of the nerve.

Five days before the ligature came away, the patient complained of an increase of pain in the wound; and, although the skin was not inflamed, considerable thickening of the subjacent parts, accompanied with tenderness, was to be detected upon pressure. These symptoms diminished upon substituting a poultice for the light dressing which had been previously used, and applying a few leeches.

---

### *Exostosis of the First Rib, with strong Pulsation of the Subclavian Artery.*

John Reynard, ætat. fifty-eight, was admitted into the Middlesex Hospital, on the 19th of May, under the care of Mr. Mayo: he had already attended several weeks as an out-patient.

In January last, he began to feel pricking and thrilling sensations, which extended from the shoulder to the hand on the right side: these he attributed to having walked several miles a short time before, with a load, which he carried principally upon the right shoulder.

There is a prominent and pulsating tumor above the right clavicle, in the situation of the subclavian artery. Below the clavicle, the artery may be felt pulsating with ordinary force, through half the extent of the axilla: at this point the artery has seemingly become obliterated. No pulsation is to be felt in any artery of the right arm, below the middle of the axilla.

After several careful examinations of the pulsating tumor in the neck, Mr. Mayo is of opinion that it consists of an exostosis, of the size of a walnut, growing from the first rib. This exostosis has raised the artery, and thrown it forward. The artery is flattened by the pressure of the tumor, so as to present an unusual breadth, but it does not seem to be enlarged.

The thrilling sensations are felt only when the arm is moved, or when, in examining the tumor, the axillary nerves are compressed: these sensations, as produced by occasional motions of the hand and arm, have latterly rather diminished than increased; it is, therefore, probable that the exostosis is not enlarging. The patient's general health is good.

### *Stone in the Bladder: Lithotomy.*

James Shuffle, ætat. nineteen, was admitted into the Middlesex Hospital, under the care of Mr. Mayo, on the 12th of April: he had laboured under the usual symptoms of stone in the bladder during several years. On sounding him, a single stone was felt, which was thought to be not larger than a chesnut. As the bladder was by no means irritable, Mr. Mayo thought the case a favorable one for the operation of lithotrity; but two attempts, which he made, were unsuccessful. In the first, the stone was seized without much difficulty, but, from an imperfection in the instrument, the drill made no impression upon it. The instrument employed on this occasion was made by Messrs. Weiss; the drill was turned by means of a winch handle. In the second attempt, Mr. Mayo used the same instrument, but with a drill bow: the stone was seized with great care, and the drilling process commenced, but in a few seconds the stone escaped from the instrument, and the patient, who was singularly timorous, expressed strongly his wish to be cut, instead of undergoing a continuance of the lithotritic process.

Accordingly, on the 3d of June, the lateral operation was performed: a stone, of the size of a walnut, was extracted, with several fragments which had been broken from it in the previous attempts at lithotrity. The operation was performed with great celerity; no untoward symptom occurred; and, before a fortnight had elapsed, the patient again passed the urine entirely by the natural passage.

The patient says that he suffered less in the operation of lithotomy, than in the previous attempts to pulverise the stone: he states, at the same time, that the introduction of the staff was the worst part of the lateral operation.

---

### *Cases of Disease of the Subcutaneous Bursa of the Patella.*

In Northumberland ward, Middlesex Hospital, there are three patients, women, between twenty and forty years of age, with diffeent affections of the bursa of the patella: they were admitted on the 23d of May, under Mr. Mayo's care.

In one of these, the bursa upon the right patella was converted into a solid, opaque, membranous structure, varied at one part by containing a small quantity of whitish substance, like firm curd. This little tumor was circular, an inch and a half in diameter, and half an inch in thickness at the middle: it was removed, and found not to adhere very closely, either to the skin or to the tendinous sheath of the patella. The wound healed readily.

In the second of the three cases referred to, at the time of the patient's admission, the bursa was swollen with fluid, and extremely painful; the skin red; and the leg and half of the thigh affected with phlegmonous erysipelas, which principally occupied the outside of the limb, and had followed the inflammation of the bursa: with this there was present considerable irritative fever, for which appropriate medicines were given. At the same time Mr. Mayo opened freely the inflamed bursa, and made another incision, three inches in length, through the most tumid and brightest part of the inflamed limb. Poultices and fomentations were afterwards prescribed. The patient recovered rapidly: the bursa discharged a thin pus for several days, and then healed.

The third of the cases referred to was of the common description: the bursa very slightly thickened, but distended with fluid, uneasy, and painful, the most ordinary state of the *housemaid's* knee. The patient being in good health, the knee was kept at rest for two days, with a cold lotion applied to it. The bursa was punctured on the third day, the fluid discharged, the wound closed, and a bandage, moistened with a cooling lotion, applied. A few days afterwards, the fluid, having reaccumulated, was again let out through a small lancet puncture, the bandage and lotion of muriate of ammonia reapplied. The fluid has not reaccumulated, nor is there any pain or tenderness of the part.

This method of treatment, which is the surest, and perhaps the best, mode of curing the complaint, is equally applicable to those cases of distended bursæ which contain, in addition to the usual fluid, clusters of soft bodies resembling melon seeds.

